# Advances in computational and translational approaches for malignant glioma

**DOI:** 10.3389/fphys.2023.1219291

**Published:** 2023-06-19

**Authors:** Adip G. Bhargav, Joseph S. Domino, Anthony M. Alvarado, Chad A. Tuchek, David Akhavan, Paul J. Camarata

**Affiliations:** ^1^ Department of Neurological Surgery, University of Kansas Medical Center, Kansas City, KS, United States; ^2^ Department of Neurological Surgery, Rush University Medical Center, Chicago, IL, United States; ^3^ Department of Radiation Oncology, University of Kansas Medical Center, Kansas City, KS, United States; ^4^ Department of Cancer Biology, University of Kansas Medical Center, Kansas City, KS, United States; ^5^ Bioengineering Program, University of Kansas Medical Center, Kansas City, KS, United States

**Keywords:** glioma, diagnostics, personalized medcine, modeling, heterogeneity, therapeutics, artificial intelligence

## Abstract

Gliomas are the most common primary brain tumors in adults and carry a dismal prognosis for patients. Current standard-of-care for gliomas is comprised of maximal safe surgical resection following by a combination of chemotherapy and radiation therapy depending on the grade and type of tumor. Despite decades of research efforts directed towards identifying effective therapies, curative treatments have been largely elusive in the majority of cases. The development and refinement of novel methodologies over recent years that integrate computational techniques with translational paradigms have begun to shed light on features of glioma, previously difficult to study. These methodologies have enabled a number of point-of-care approaches that can provide real-time, patient-specific and tumor-specific diagnostics that may guide the selection and development of therapies including decision-making surrounding surgical resection. Novel methodologies have also demonstrated utility in characterizing glioma-brain network dynamics and in turn early investigations into glioma plasticity and influence on surgical planning at a systems level. Similarly, application of such techniques in the laboratory setting have enhanced the ability to accurately model glioma disease processes and interrogate mechanisms of resistance to therapy. In this review, we highlight representative trends in the integration of computational methodologies including artificial intelligence and modeling with translational approaches in the study and treatment of malignant gliomas both at the point-of-care and outside the operative theater *in silico* as well as in the laboratory setting.

## 1 Introduction

Cancer mortality continues to rise as management of other chronic diseases improves (ReFaey et al., 2021). Within brain cancer, gliomas comprise the most common subset of primary malignant brain tumors in adults (Ostrom et al., 2021). These tumors are uniformly fatal for the vast majority of patients afflicted with this disease process despite current standard-of-care therapy typically including maximal, safe cytoreductive surgery and a combination of chemotherapy and radiation therapy tailored to the grade and genetic makeup of the subtype of glioma in question (Sanai and Berger, 2018; Molinaro et al., 2020; Hervey-Jumper et al., 2023). For high-grade gliomas including glioblastoma, the cornerstone adjuvant therapy continues to be the Stupp protocol (Stupp et al., 2005). Though improvements in long-term survival have been observed with advances in surgical technique and chemotherapy in low-grade gliomas, prognosis remains dismal overall and particularly devastating in patients harboring high-grade gliomas (Sanai and Berger, 2018; Ostrom et al., 2021).

Investigations into the tumorigenesis and growth of gliomas as well as factors contributing to treatment failure over recent years have underscored several disease features that may be implicated in the refractory nature of the disease. It is now clear that within glioblastoma there is significant heterogeneity at multiple levels of the disease process that may affect response to therapies (Bonavia et al., 2011; Saygin et al., 2019; Garcia et al., 2021; Mitchell et al., 2021). These include heterogeneity at the cellular and molecular level within the tumor and locoregional heterogeneity in different regions of the tumor and surrounding microenvironment. In addition, there is suspected to be a component of heterogeneity in response to therapy at a patient and population level that may be influenced by a complex interplay of comorbid medical conditions, medications, and other patient factors that is poorly understood (Gimple et al., 2019; Saygin et al., 2019; Mitchell et al., 2021). Plasticity and dynamic evolution inherent to glioblastoma further complicates the development of targeted therapies due to a subset of stem-like cells within the tumor referred to as glioma stem cells or brain tumor-initiating cells (BTICs) which have been shown to play a key role in treatment resistance and ultimately disease progression (Saygin et al., 2019; Prager et al., 2020; Andersen et al., 2021). This subset of cells leverages various mechanisms at the cellular and molecular level including resistance mechanisms and enhanced migration to produce differential responses to current therapies and enable tumor recurrence (Gimple et al., 2019; Bhargav et al., 2020; Prager et al., 2020; Garcia et al., 2021).

Overcoming practical limitations of surgical and medical therapies and improving clinical decision-making as to when and which therapies to administer has been an area of growing interest in light of novel technologies. With regards to surgical therapies, extent of resection has been historically limited by the ability of the neurosurgeon to accurately differentiate between normal brain and glioma in order to determine whether the boundaries of the resection may be pushed safely. Moreover, given the invasive nature of gliomas and consequent spread into eloquent regions of brain crucial for preserving acceptable quality of life, groups have also focused efforts on developing models to better define glioma-network boundaries to optimize onco-functional balance (Duffau and Mandonnet, 2013; Lara-Velazquez et al., 2017; Sanai and Berger, 2018). In this review, we highlight disruptive point-of-care as well as systems- and laboratory-level methodologies and advances as an update on emerging trends in the study and treatment of gliomas.

## 2 Integrated point-of-care methodologies

**FIGURE 1:**
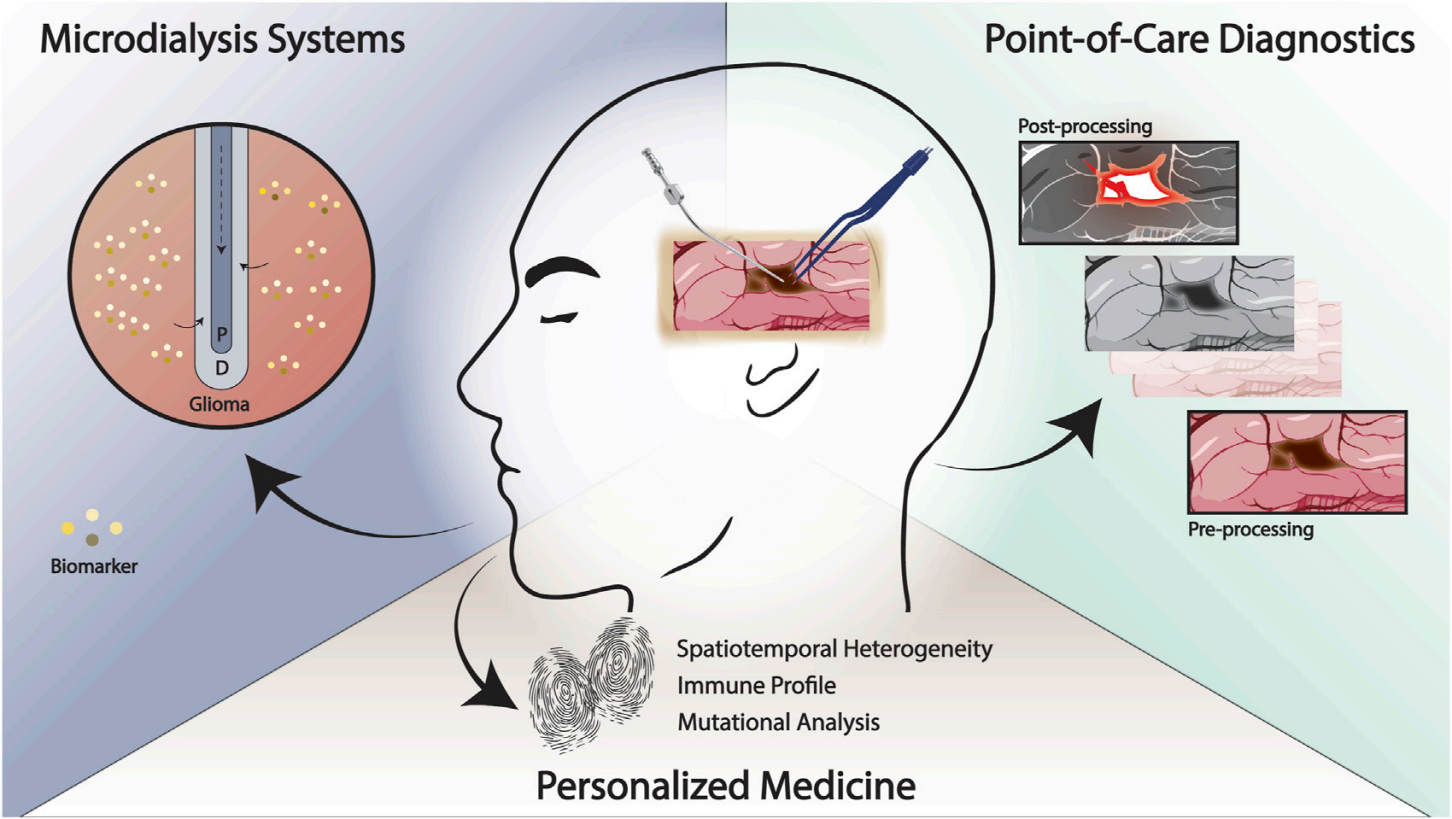
Emerging methodologies for the study and treatment of glioma at the point-of-care. Rapid, intraoperative techniques that enable improved delineation of the tumor margin and patient-specific “fingerprinting” may facilitate the development of effective therapies. Intraoperative experimental paradigms such as real-time microdialysis has the potential to identify patient-specific biomarkers and improve our understanding of treatment response and recurrence. P, perfusate; D, dialysate.

A longstanding obstacle in the development and translation of effective therapeutics for glioma has been in part, achieving a high-fidelity recapitulation of the human disease process and microenvironment. Although laboratory modeling of glioma has continued to evolve in sophistication and accuracy, a true understanding of disease features at a molecular, cellular, and tissue level is lacking (Gomez-Oliva et al., 2020; Garcia et al., 2021; Mariappan et al., 2021). As a result, in recent years, efforts have been directed towards devising strategies to utilize the operating room as an extension of the laboratory for both mechanistic study of glioma pathophysiology and diagnostics summarized in Figure 1.

Understanding *in situ* metabolic processes and response to therapeutics in real-time has been a goal of investigation but has been limited by disease modeling. A decade’s-old paradigm is currently being revisited in this regard with the use of intraoperative microdialysate systems for real-time data capture at the time of surgery and in the immediate postoperative period (Roslin et al., 2003; Marcus et al., 2010; Wibom et al., 2010; Goodman, 2011). Previously, groups have employed cerebral microdialysis to simultaneously deliver chemotherapeutic agents and to assess therapeutic response via select biomarkers as well as to identify markers of response to adjuvant therapies (Roslin et al., 2003; Wibom et al., 2010; Goodman, 2011; Bjorkblom et al., 2020; Pierce et al., 2021). *Björkblom et al.* treated ten patients with recurrent glioma after second-line chemotherapy had been exhausted using an implanted microdialysis catheter that administered cisplatin (Bjorkblom et al., 2020). Serial samples were obtained of the interstitial fluid and serum that identified correlation of survival with certain response patterns including low levels of lactic acid, glyceric acid, and cysteine in tumor samples and low levels of ketohexoses and glycerol-3-phosphate in serum samples (Bjorkblom et al., 2020). *Wiborn et al.* use a similar paradigm to identify characteristic metabolomic patterns in patients with glioblastoma undergoing radiation therapy (Wibom et al., 2010). Ongoing clinical trials (NCT04692337, NCT04047264) aim to utilize a similar paradigm but with the incorporation of real-time, *in situ* and subsequent rapid assessment of potential therapeutic biomarkers and metabolomics. Advances in microelectrode and catheter technology including the development of lower profile instruments as well as those that can simultaneously sample brain interstitial fluid and neural activity hold promise for a more integrated, real-time assessment of molecular- and network-level changes in gliomas (Rajani et al., 2021; Stangler et al., 2023). *Stangler et al.* demonstrate that an integrated microperfusion-EEG electrode is able to detect epileptiform activity as well as sample interstitial fluid in a pig model with potential future implications of studying glioma-neuronal integration and functional reorganization *in situ* (Stangler et al., 2023).

Several novel point-of-care diagnostic methodologies have also been developed in recent years with the goal of providing real-time, clinically actionable feedback to the neurosurgeon which has historically not been possible (Ji et al., 2013; Kut et al., 2015; Shankar et al., 2015; Orringer et al., 2017; Pirro et al., 2017; Juarez-Chambi et al., 2019). The need for rapid and accurate diagnosis is particularly important in the management of diffuse gliomas where the margin between normal brain and tumor-invaded tissue is increasingly obscured or in situations where the differential diagnosis based on preoperative evaluation and imaging is equivocal. *Kut et al.* developed a methodology to use optical coherence tomography (OCT), which has previously been used in other clinical settings including ophthalmology to delineate glioma tissue from normal brain *in vivo* and *ex vivo* brain tumor models (Kut et al., 2015). In subsequent studies, this group incorporated artificial intelligence-assisted models including one that employs deep convolutional neural networks using intraoperatively obtained human brain and glioma specimens to further validate the clinical translation potential of this approach (Juarez-Chambi et al., 2019; Wang et al., 2023). In one study, they demonstrate 100% sensitivity and 85% specificity thresholds in detecting glioma-infiltrated tissue as well as significantly increased processing speeds compared to prior iterations of the technology (Juarez-Chambi et al., 2019). The authors note that this initial computational framework exhibited a misclassification rate of approximately 15% and acknowledge that future modifications may need to incorporate a more sophisticated algorithm that accounts for glioma subtypes (Juarez-Chambi et al., 2019). Though this technology holds promise, in-human, real-time testing is yet to be conducted and will also require optimization of a handheld OCT probe that may be used intraoperatively (Juarez-Chambi et al., 2019). Similarly, in their initial study, *Ji et al.* demonstrate the utility of a novel imaging technique, stimulated Raman scattering (SRS) microscopy was able to delineate brain tumor-infiltrated tissue from normal brain tissue in *vivo* and *ex vivo* xenograft murine models of glioma as well as in human surgical specimens (Ji et al., 2013). *Orringer et al.* later developed a portable microscope with the capability of performing SRS microscopic analysis intraoperatively (Orringer et al., 2017). In a cohort of 101 neurosurgical patients, they report κ > 0.89 concordance of SRS microscopy and histopathologic diagnosis as well as 90% accuracy in delineating tumor subtypes (Orringer et al., 2017). *Hollon et al.* further optimize this technology in combination with an artificial intelligence-based screening workflow, DeepGlioma, to classify the molecular subtypes of diffuse glioma (Hollon et al., 2023). They report that this screening test, validated through a multicenter, prospective, international patient cohort undergoing SRS imaging, predicted the WHO molecular classification of diffuse gliomas with an accuracy of 93.3% in under 90 s (Hollon et al., 2023).

In addition to diagnostic applications, other point-of-care methodologies aim to characterize heterogeneity in gliomas to enable true personalized medicine approaches to therapy. As aforementioned and discussed in detail elsewhere (Saygin et al., 2019; Lathia et al., 2020), it is well known that one of the key challenges in the development of successful targeted therapies for glioma lies in the multiple layers of heterogeneity inherent to the disease process which encompasses the genetics and epigenetics, metabolic profile, and immune landscape, among others (Gimple et al., 2019; Bhargav et al., 2020; Garcia et al., 2021; Himes et al., 2021). All of these differences have been shown to have a significant impact on response to therapy in prior studies (Gimple et al., 2019; Prager et al., 2020; Comba et al., 2021; Medikonda et al., 2021). Several methods have emerged in the last few years that include the use of mass spectrometry, liquid biopsy, or other high-throughput assays with the potential for rapid intraoperative application and “fingerprinting”. In a series of studies led by Cooks, a proof-of-concept high-throughput methodology is validated for rapid intraoperative molecular classification of gliomas using desorption electrospray mass spectrometry whereby an intraoperative smear of tumor tissue can be analyzed and yield accurate output of molecular tumor characteristics (Pirro et al., 2017; Chen et al., 2021; Morato et al., 2022). This is achieved by subjecting sample-derived oncometabolites and membrane-phospholipids, in one iteration of this technology, to produce a molecular fingerprint with accuracies of >85% and total assay time of <5 min (Chen et al., 2021). Groups have also worked to optimize older technologies such as polymerase-chain reaction-based assays to characterize molecular characteristics of gliomas in an intraoperative setting and in a clinically actionable timeframe (Shankar et al., 2015; Djirackor et al., 2021; Muralidharan et al., 2021). Liquid biopsy remains a significant area of active investigation as a means to monitor glioma response to therapy at the point-of-care which may be limited by lack of specificity and low-mutational burden observed in a certain subset of gliomas (Bettegowda et al., 2014; Miller et al., 2019). *Panditharatna et al.* develop a platform that utilizes patient-derived plasma and cerebrospinal fluid samples from which circulating tumor DNA is extracted (Panditharatna et al., 2018). They use a multiplex platform to detect driver mutations in patients with diffuse midline glioma undergoing treatment to assess treatment response and disease progression (Panditharatna et al., 2018). They demonstrate that the plasma-based detection of the H3K27M mutation correlated with treatment response to radiation therapy on MRI (Panditharatna et al., 2018). Similar platforms are being developed for adult diffuse gliomas (Panditharatna et al., 2018). A detailed overview of this technology and potential hurdles to clinical application can be found elsewhere (Mair and Mouliere, 2022). Recent evidence has also highlighted the critical role of the glioma microenvironment and immune landscape with regards to therapy resistance and the need to characterize this aspect of the disease process in order to improve the efficacy of immunotherapies (Fecci and Sampson, 2019; Himes et al., 2021; Medikonda et al., 2021). To this end, groups have sought to develop a workflow at the point-of-care to identify personalized neoantigen profiles to develop patient-specific vaccination strategies (Johanns et al., 2019; Dunn et al., 2020; Dunn et al., 2022; Schaettler et al., 2022). *Johanns et al.* describe the treatment of a patient with glioblastoma where the group’s immunogenomics pipeline for neoantigen determination and characterized reviewed in detail by *Dunn et al.* was implemented as a proof-of-concept (Johanns et al., 2019; Dunn et al., 2020; Dunn et al., 2022).

## 3 Novel modeling approaches of systems-level processes

Beyond advances in understanding the biological basis of glioma disease progression and recurrence at the molecular, cellular, and tissue level, new evidence suggests that gliomas also leverage systems-level processes (Hadjiabadi et al., 2018; Stoecklein et al., 2020; Young et al., 2020; Krishna and Hervey-Jumper, 2022; Krishna et al., 2023; Winkler et al., 2023). In recent years, this has led to the development of entirely new fields of study including cancer neuroscience to better understand the implications of glioma-induced systems-level changes in the human brain (Winkler et al., 2023). Progress in this realm has been historically hindered by the inadequacy of computational models to capture systems-level dynamics, and this continues to be an ongoing challenge. In this section, we briefly highlight recent advances in our understanding of these processes and the tools that have enabled us to do so with an eye towards future directions.

The study of brain connectomics in the context of glioma has gained traction recently due to its potential paradigm-shifting implications for both neurosurgical care and new therapies. With the completion of the Human Connectome Project, groups have developed more accurate methodologies to recreate patient-specific white matter representations of large-scale brain networks that subserve higher order function (Glasser et al., 2016; Maier-Hein et al., 2017; Hadjiabadi et al., 2018; Briggs et al., 2022; Dadario et al., 2023). The Quicktome platform is one such tool that is approved by the Federal Drug Administration for network analysis that employs machine-learning parcellation scheme of functional connectivity as opposed to previous anatomy-based schemes which may yield distorted models in the setting of glioma-infiltrated brain (Morell et al., 2022). *Morell et al.* leverage this platform in a retrospective study of patients with gliomas to characterize the impact of these tumors on large-scale networks (Morell et al., 2022). They find that certain networks such as the central executive network, default mode network, and the dorsal attention networks are affected more frequently and describe a significant correlation between preoperative deficits and network involvement (Morell et al., 2022). With this new modeling approach, groups have proposed a shift towards connectome-based glioma surgery where the extent of surgical resection is not only determined by the boundaries of tumor and surrounding tissue changes observed on neuroimaging, but also the networks and are areas of network convergence involved to maximize removal of glioma-infiltrated brain (Duffau, 2014; Sarubbo and Duffau, 2021). This approach suggests that connectome-based surgery may be crucial in preserving higher-order functions that are currently poorly understood and escapes detection of conventional methods of mapping function including brain mapping and functional imaging (Duffau, 2014; Dadario et al., 2021; Sarubbo and Duffau, 2021; Yeung et al., 2021). Though this methodology is yet to be widely adopted, emerging evidence may further inform the philosophy behind achieving oncofunctional balance through neurosurgical care of patients with gliomas.

**FIGURE 2:**
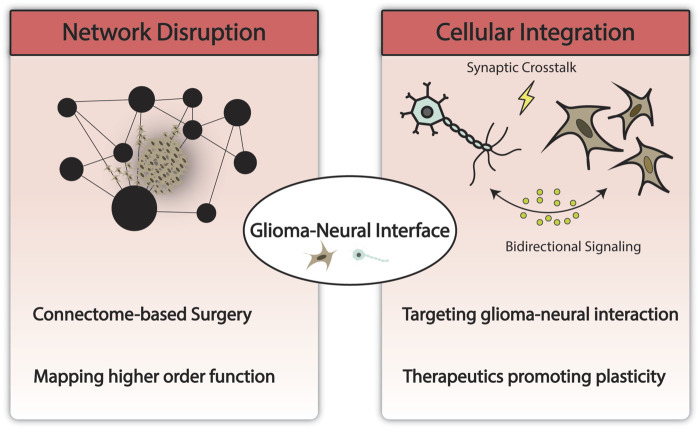
Modeling the glioma-neural interface and its implications. Technological advances in computing have enabled the modeling of large-scale brain networks and methodology to study network disruption by glioma. This may help uncover and preserve higher order functions and has the potential to impact the current surgical paradigm. At the cellular and molecular level, understanding network disruption and glioma-neural integration may yield novel therapeutics targeting neural regulation of cancer and network remodeling.

Early laboratory studies have begun to provide potential biological substrates for the aforementioned systems-level changes which may have implications for a new generation of therapeutics focused on slowing or ameliorating network disruptions and thereby improving overall functional outcome and recovery (Venkatesh et al., 2015; Venkatesh et al., 2019; Krishna et al., 2023). These studies suggest that glioma cells integrate with neural elements and brain circuitry via synaptic and paracrine crosstalk as well as the development of structural changes that enable integration into the surrounding microenvironment and networks (Venkatesh et al., 2015; Venkatesh et al., 2019; Krishna et al., 2023). In depth discussion of these emerging mechanisms can be found in excellent reviews by Krishna and Hervey-Jumper (2022) and Winkler et al. (2023). As these mechanisms are further elucidated, preclinical modeling of gliomas and therapy development must incorporate the study of network level changes in addition to conventional models that account for traditional hallmarks of cancer and progression. Currently, high-fidelity, high-throughput preclinical models and proxies that can recapitulate patient-specific representations of glioma-neuronal integration are lacking and will be an area of future investigation. Areas of investigation and potential implications are depicted in Figure 2.

As large datasets become increasingly available and accessible ranging from radiographic data to genomic data, other subfields in mathematical oncology applied to glioma continue to evolve as well. Various modeling approaches have been successfully employed to forecast and represent different aspects of the glioma disease process including disease heterogeneity, development and evolution of treatment resistance, growth, and invasion (Rockne et al., 2019). *Tripathi et al.* develop a mathematical model that characterizes the invasion profile of IDH-wild-type glioblastoma in a cohort of 101 patients which suggests theoretical threshold for benefit of supramarginal resection (Tripathi et al., 2021). They note that supramarginal resection of tumors quantified as moderately or highly diffuse in their model is significantly correlated with overall survival and observe a lower threshold-based benefit for supramarginal resection in nodular tumors (Tripathi et al., 2021). Similar radiomics and radiogenomics methodologies have been used to characterize spatial heterogeneity in gliomas that incorporates predictions of cell density, growth kinetics, and growth patterns with implications of patient counseling, patient-specific adjuvant therapy plans, and surgical planning (Hu et al., 2015; Hu et al., 2017; Yang et al., 2019; Hu et al., 2020; Massey et al., 2020; Whitmire et al., 2020).

## 4 Developments in laboratory-based modeling and therapy development

The emergence of new concepts in our understanding of glioma heterogeneity and mechanisms of resistance has necessitated increased sophistication of preclinical modeling. In particular, improved understanding of sex differences, evolutionary dynamics and plasticity of BTICs, and the robust immune landscape inherent to glioma biology has driven advances in laboratory modeling (Yang et al., 2019; Garcia et al., 2021; Himes et al., 2021; Mitchell et al., 2021). *Garcia et al.* describe a methodology for the functional characterization of BTICs which involves the creation of orthotopic murine models in a sex-specific manner (Garcia et al., 2021). In addition, they describe several high-throughput *in vitro* assays that can be used to characterize features of BTICs that are known to affect survival such as markers of stemness, response to novel therapeutics, and migration capacity (Garcia et al., 2021). Still, there exists a gap between laboratory-based interrogation of glioma in animal models and translating these findings with reliability. The majority of existing preclinical models lack validation in patients which persists as a major limitation. *Wong and Shah et al.* develop a microfluidic device that addresses this gap with regards to the migration of BTICs (Wong et al., 2021). In this study, they subject patient-derived BTICs to a migration assay using a microfluidic device and show that quantitative measurement of *in vitro* migration as well as proliferation capacity can be used to predict progression-free survival with 86% accuracy in a retrospective cohort of 28 patients (Wong et al., 2021). Other groups have also developed microfluidic, tumor-on-a-chip systems to interrogate drug efficacy as well as novel drug delivery approaches including nanoparticle-based therapies (Fan et al., 2016; Xiao et al., 2019; Chen et al., 2020; Liu et al., 2021). The latter employs techniques such as surface plasmon resonance that can assess target-specificity and binding interactions (Schneider et al., 2015; Bhargav et al., 2020). Nevertheless, such systems suffer from the same limitation. Moreover, the use of conventional culture-based systems cannot accurately model intratumoral heterogeneity as a single clonal population of BTICs is selected in the culture process (Lathia et al., 2020; Mitchell et al., 2021). *Jacob et al.* recently developed a patient-derived organoid model of glioblastoma requiring minimal processing that recapitulates heterogenous cell populations which may offer higher fidelity therapy interrogation (Jacob et al., 2020). They note that with their process, the cytoarchitecture of the surgical sample is also minimally disrupted which may aid in maintaining the mechanical properties of the tumor–another component of glioma heterogeneity that may impact malignancy (Jacob et al., 2020). *Abdullah et al.* achieve a similar model of low-grade glioma which holds promise for more nuanced drug development (Abdullah et al., 2022).

## 5 Discussion

With recent discoveries in glioma biology, glioma-network integration, and heterogeneity, more questions than answers are generated and the challenge of developing effective therapies persists; however, the evolution of computational technologies and computing capacity has opened several doors to tackle this challenge. Artificial intelligence, mathematical modeling, and analysis of large, often patient-specific data sets will be a critical component of methodologies in studying and treating glioma. The representative trends and advances described in this review hold great promise to disrupt existing paradigms in disease modeling and clinical translation.
